# Another Contributory Scheme

**Published:** 1922-10

**Authors:** Charles Oakey

**Affiliations:** Secretary, Billingsgate Christian Mission & Dispensary, 19 St. Mary-at-Hill, E.C.3.


*To the Editor of* THE HOSPITAL AND HEALTH REVIEW.

### Another Contributory Scheme.

*To the Editor of* THE HOSPITAL AND HEALTH REVIEW.

SIR,—In connection with the schemes now under consideration to provide a *certain income* for hospitals in the metropolitan area particularly, may I state that I have submitted the following suggestions to King Edward's Hospital Fund :—

Each hospital should collect in its *own* area or district (after defining boundaries) a voluntary individual donation, say, 3d. A willing unpaid collector should be found in each thoroughfare, preferably a baker, chemist, or confectioner, &c., who, to save book work, should simply be provided with a receipt book from which he would give a receipt, entering the number of disc or card on the counterfoil, with date. The monies collected should be pooled periodically, and divided between hospitals according to the cost and number of beds occupied by "Weekly Donors" during the period (say, quarterly or half-yearly); any surplus to be shared by all. Of course, there would be a 'phoning of other hospitals for vacant beds should they be required. Uniformed nurses (or, if nurses could not be spared, junior clerks) should collect weekly or fortnightly from shopkeepers the monies and counterfoil books, leaving a temporary receipt book for use. Shopkeepers should be provided with a simple form, in the *first instance* for applicants, with *two* items only thereon: (1) Employers (or neighbouring householders where there is "no occupation"), certifying as to the salary or wages limit; (2) certifying as to number and ages of children in family, on statement of applicants. If these prove satisfactory, a small numbered metal disc, or card, should be handed to the applicant, on next visit. A "record" of numbers, names and addresses with columns for payments should be kept by all hospitals, covering completely their own districts.

Secretaries of neighbouring general hospitals should meet informally and agree to boundaries. £10 or £20 would easily cover receipt books, number discs or cards and simple proposal forms.

CHARLES OAKEY,
Secretary, Billingsgate Christian Mission & Dispensary, 19 St. Mary-at-Hill, E.C.3.

### MR. ORDE'S SUCCESSOR AT NEWCASTLE.

AT a meeting of the House Committee of Newcastle Royal Victoria Infirmary on Sept. 7, Mr. R. H. P. Orde, late secretary and house governor, who is retiring after 22 years' service, to take up work on the Joint Council of the British Red Cross and St. John of Jerusalem, was presented with a cheque for £162, and a solid silver coffee service. We need not remind our readers what a loss the Royal Infirmary has suffered in the departure of Mr. Roden Orde. His record there is long and distinguished, and entailed an immense amount of work, administrative no less than financial, in the growth of workmen's subscriptions and the unwieldy size of the committees to which they often give rise. Hospital life is rich in proportion as such men as Mr. Orde control its administration, and he has set a standard which must inspire his former colleagues and successors.

The vacancy has been filled by the appointment of Mr. Sydney Dunstan. The salary is £700 a year. Mr. Dunstan, who was chosen out of 80 applicants, has been for 16 years in charge of the dispensary and stores department at the Royal Infirmary. He first came to Newcastle from the London Hospital, prior to which he was engaged in the wholesale drug trade. His main concern at Newcastle has hitherto been in the purchase and distribution of drugs and stores, instruments and dressings, and the bakery and laundry have been in his charge. It is becoming the practice to divide the work of the secretarial departments into finance and administration, with a separate officer in charge of each, and the result is that the term "secretary" is less precise than it used to be, and that the qualifications for a post in one institution may not be precisely those required when another of the same nominal description is vacant elsewhere. We offer to Mr. Dunstan our warm congratulations upon his promotion.

